# Cisplatin triggers cancer stem cell enrichment in platinum-resistant cells through NF-κB-TNFα-*PIK3CA* loop

**DOI:** 10.1186/s13046-017-0636-8

**Published:** 2017-11-23

**Authors:** Bhushan Thakur, Pritha Ray

**Affiliations:** 1 Imaging Cell Signaling and Therapeutics Laboratory, Advanced Centre for Treatment, Research and Education in Cancer (ACTREC), Tata Memorial Centre, Navi Mumbai, India; 2 Homi Bhabha National Institute, Mumbai, Anushakti Nagar India

**Keywords:** CSC-enrichment, PIK3CA regulators, NF-κB-TNFα-*PIK3CA* loop

## Abstract

**Background:**

Parallel to complex alteration in molecular and cellular events, enrichment of cancer stem cells (CSC) contributes significantly in deliberation and maintenance of cisplatin resistance. Cisplatin mediated CSC enrichment is well established in various cancers, yet the underlying mechanism is largely unknown. Cisplatin also promotes transcriptional upregulation of *PIK3CA*, hence activating PI3K/AKT signaling in resistant cells. However, such cisplatin-induced transcriptional regulators of *PIK3CA* and their impact on cancer stem cell population in resistant cells are largely unknown.

**Methods:**

DNA-binding protein pulldown using *PIK3CA* promoter as bait followed by nLCMS was used to identify, cisplatin-induced potential transcriptional regulators of *PIK3CA* promoter. *PIK3CA* promoter activity was estimated by luciferase based reporter assay. ChIP was used to assess interaction of NF-κB with *PIK3CA* promoter. CSC-enriched side-population was sorted using DCV-dye exclusion methods. All the gene expression levels were assessed using qPCR.

**Results:**

Using a transcription factor pull-down assay with *PIK3CA* promoter, we identified NF-κB as a prime regulator, which escalates both *TNFα* and *PIK3CA* expression only in CSC enriched side-population (SP) but not in non side-population (NSP) in platinum resistant ovarian cancer cells upon cisplatin treatment. This SP-specific NF-κB-TNFα-PIK3CA bi-modal loop, on one hand, maintains persistent activation of NF-κB through TNFα- NF-κB autocrine loop, while NF-κB-PIK3CA loop nurture CSC population under cisplatin treatment. Activation of PI3K/AKT signalling drives SP’s into an undifferentiated, anti-apoptotic stage through upregulating *P21*, *P27,cFLIP* expression. Contrarily, lack of active NF-κB-TNFα-PIK3CA loop makes NSPs vulnerable towards cisplatin and undergoes apoptosis. Altogether, cisplatin enriches cancer stem cells properties in SP fraction, which is evident from increased levels of pluripotency gene *OCT4*/*SOX2*/*NANOG* expression. Disruption of *PIK3CA*-NF-κB loop by Wortamannin reduces SP fraction by 1.4–1.6 fold in control and treated cells.

**Conclusion:**

Together, our study signifies an active role of NF-κB-TNFα-PIK3CA bi-modal loop in cisplatin-mediated promotion and maintenance of CSC-like population in platinum-resistant cells.

**Electronic supplementary material:**

The online version of this article (10.1186/s13046-017-0636-8) contains supplementary material, which is available to authorized users.

## Background

Chemotherapy is one of the prevailing methods to manage neoplastic growth. Unfortunately, generation of resistance substantially handicaps the efficacy and results in significant mortality in cancer patients. While molecular alteration in signaling cascades aid in acquirement and maintenance of resistance, a small fraction of inherently resistant cancer stem cells (CSC) help in repopulating the chemoresistant tumor [1–3]. In particular, these drug resistant CSC’s evolve to resist therapy setbacks resulting in incessant growth and relapse. Hence targeting the CSC component has a great therapeutic potential in therapy-resistant disease. However, theoretically achievable, such objective is extremely challenging and requires in depth understanding of how CSC’s response towards chemotherapeutics.

CSC’s are a small fraction of heterogeneous tumor population identified by surface markers like CD34+/CD38- (AML), ESA+/CD44+/CD24-(low) (breast cancer) and functional properties such as side-population (SP) or aldehyde dehydrogenase activity [1]. Though association between CSCs and chemoresistance is well established, the key molecular events involved in the regulation of CSCs remain largely unknown. Till date, differential activation of PI3K/AKT, WNT, NOTCH and NF-κB signaling are linked to maintenance of CSC phenotype and chemoresistance [4–6]. However, the actions and the outcomes of cancer therapeutics on signaling cascades in CSCs still remain poorly understood.

Cisplatin, a DNA damaging agent, also modulates several signalling cues including c-ABL, p53 signaling, MAPK/JNK/ERK, PI3K/AKT, NF-κB-signalling, FAK and WNT-signaling [7]. Cisplatin resistance is a net effect of multiple mechanisms that either inhibit apoptosis, promote cell survival, or both. Amongst these, the nuclear Factor-kappa B (NF-κB) has been identified as a key player in platinum resistance [8, 9]. A variety of stimuli coalesces on NF-κB activation, which mediated upregulation of *cFLIP*, *BCl-XL, XIAP* and favours survival of cisplatin resistant cells [8, 9]. NF-κB also prevents cisplatin mediated histone acetylation and BRCA1 nuclear translocation in HNSCC and inhibit cisplatin cytotoxicity [10]. In response to extracellular signals, a number of RTKs can activate NF-κB via PI3K/AKT or JNK/STAT pathway [11]. In many cancers, hyperactivation of PI3K/AKT, a key survival pathway contributes to tumor growth, metastatic competence, and therapy-resistance [12]. Besides RTK mediated activation, PI3K gene is regulated by few central transcription factors (p53, NF-κB, FOXO3a, YB1) in chemosensitive cells [13], however, regulators of *PIK3CA* in chemoresistant cells particularly in response to drugs has never been identified. We have been investigating the underlying mechanism of upregulated PI3K expression in platinum-resistant cells, and demonstrated that in absence of a cisplatin-induced Ser46-phosphorylation, p53 failed to bind and represses *PIK3CA* promoter leading to activation of PI3K/AKT signalling that actively sustained survival but not proliferation of resistant cells [14]. Though lack of PI3K promoter attenuation by cisplatin in resistant cells is indicative of loss of p53’s repressive action, it does not explain the observed increase in *PIK3CA* expression and points toward a second level of regulation for this critical gene and associated signalling.

In the present study, we identified cisplatin responsive potential transcriptional activators of *PIK3CA* in resistant cells and explored the consequence of this intricate regulation in preserving resistance and CSC-characteristics. Through DNA-protein pulldown, NF-κB was identified as a key cisplatin responsive transcription factor, which escalated *PIK3CA* specifically in CSC-enriched SP cells and governed an anti-apoptotic, dormant state. Lack of drug-induced quiescence in non-CSC fraction attributed to their susceptibility towards cisplatin. Cisplatin induced an intricate bi-modal feedback loop between TNFα-NF-κB & NF-κB-PI3K signalling leading to maintenance of CSC-phenotype. This is probably the first mechanistic report on how cisplatin drives enrichment of CSCs in platinum-resistant cells thereby favouring a tilt towards resistance maintenance.

## Methods

### Cell lines and treatment

A2780 (DMEM), A2780-CisR (DMEM), OAW42 (MEM), SKOV3 (RPMI) and TOV21G (RPMI) cells were cultured in the respective media supplemented with 10% fetal bovine serum (HiMedia) and 1% penicillin-streptomycin (GIBCO). Treatments of Cisplatin (10 μg/ml), TNFα (50 ng/ml), Forskolin (10 μM), LiCl (40 mM) and wortmanin (50 μM) were given for 24 h.

### DNA-protein pulldown and nLC-MS analysis

OS4-*PIK3CA* deletion construct (340 bp) was biotinylated by PCR amplification and DNA-protein pulldown was performed using streptavidin-coupled Dynabeads as described earlier [15]. DNA bound protein fraction was eluted with 0.1% SDS solution and was digested with trypsin (in-solution method) and subjected to Triple TOF 5500+ nano-scale liquid chromatographic tandem mass spectrometry (nLC-MS/MS) for acquiring peptide spectra.

### Data analysis and candidate selection

Significant protein candidates from each MS/MS spectra were identified using protein pilot software at 0.01% false discovery rate and *p* = 0.05 significance level. From each run, identified proteins were categorised based on GO-‘biological’ and ‘molecular’ functioning with GSEA server (http://software.broadinstitute.org/gsea/index.jsp). Only protein signatures associated with transcription factors and associated protein were used for gene family overlap and functional overlap between control and cisplatin treated samples were computed through Venn 2.1.0 server. List of identified proteins is given in Additional file 1: Table S1-S3.

### Chromatin immune precipitation (ChIP)

ChIP was performed as described earlier with few modifications [14]. Briefly, 50 μg of crosslinked-chromatin were precipitated with 2 μg of NF-κB-specific antibody and DNA was eluted post reverse-crosslinking. ChIP-DNA was analysed with semi-quantitative PCR using whole length *PIK3CA* promoter primers (primer sequences adapted from [16]). Non-immuno-precipitated chromatin was used as total input control.

### Isolation of side population

Side and non-side population cells are sorted as described earlier [17]. Membrane drug transporter inhibitor Verapamil (50 μM, Sigma) was used as negative control for gating. Data analysis was performed using DIVA software.

### Statistical analysis

All the quantitative data represents the mean ± SEM of at least three independent biological experiments and statistical significance were analysed using unpaired Student’s t test. *P* value ≤ 0.05 was considered as significant. Additional supplementary method and materials (Additional file 8).

## Results

### Cisplatin enriched side-population fraction with augmented *PIK3CA* expression in resistant cells

**Fig. 1:**
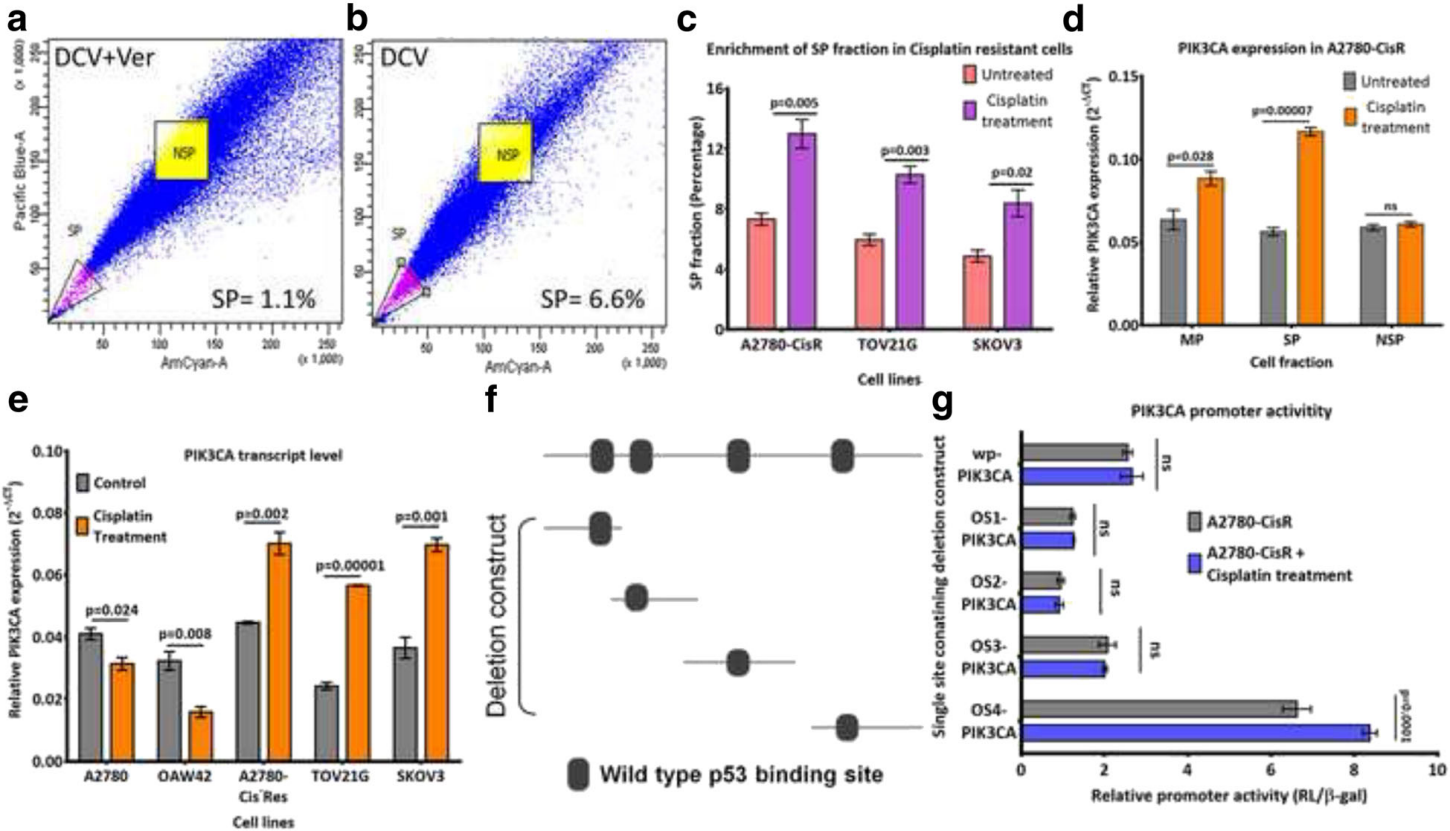
Cisplatin augmented CSC-enriched side-population (SP) and *PIK3CA* expression in resistant cells. A&B. SP of A2780-CisR represented as FACs dot plot with (**a**) and without (**b**) verapamil. c Quantification of SP in A2780-CisR, TOV21G and SKOV3 cells pre and post cisplatin treatment (*n* = 3). **d** qPCR analysis demonstrated cisplatin mediated escalation of *PIK3CA* expression only in main (MP) and SP but not in non-SP (NSP) of A2780-CisR (*n* = 3). **e** Cisplatin could induce *PIK3CA* expression only in resistant (A2780-CisR, SKOV3 and TOV21G),cells while it suppressed *PIK3CA* expression in sensitive cells (A2780 and OAW42) (*n* = 3). **f** Schematic of the full-length *PIK3CA* promoter and subsequent deletion constructs. **g** Cisplatin treatment augmented promoter activity of only OS4-*PIK3CA* construct but not OS1, 2 & 3 in A2780-CisR cells. Data were represented as average ± SEM of at least three independent replicates along with their *p*-value for statistical significance. (ns-non significant) Also see Additional file 2: Figure S1

We had earlier reported increase in CSC-enriched Side-population (SP) during acquirement of cisplatin resistance in A2780 and OAW42 cellular models. This SP fraction was associated with increased spheroid formation, upregulated pluripotent genes, and tumorigenic ability at fewer cell implantation in immune-compromised mice, the well-established characteristic features of CSCs [17]. Hence, to understand the impact of cisplatin on existing CSC-population of chemoresistant cells, we assessed the SP fraction in A2780-CisR cells pre and post cisplatin treatment. Cisplatin enhanced SP fraction from 7.3 ± 0.7% (0.65 ± 0.45% with verapamil) to 13.7 ± 2.6% (1.8 ± 0.6% with verapamil) (Fig. 1a-c). Similar cisplatin dependent increase in SP fraction was also found in other resistant cell lines like TOV21G (from 5.9 ± 0.7% to 10.2 ± 0.4%) and SKOV3 (from 4.9 ± 0.7% to 8.4 ± 1.5%) cells (Fig. 1c). These cells showed low level of cell death (15–20%) upon 24 h of sub-lethal cisplatin treatment (10 μg/ml) (Additional file 2: Figure S1A). However, drug mediated enrichment of a specific cell fraction (SP) was quite unexpected and therefore we attempted to decipher the molecular mechanism. Recent evidences revealed active involvement of PI3K/AKT/NF-κB cue in maintenance and enrichment of colon, breast, and ovarian CSCs [1]. Hence, to understand the impact of PI3K/AKT signalling in CSC, we assessed *PIK3CA* expression in main population (MP), SP, and non-SP (NSP) fraction of resistant cells pre and post cisplatin treatment. Cisplatin augmented *PIK3CA* expression in MP (1.4, 1.9 and 1.6 fold) and SP fraction (2.1, 2.2, and 1.7 fold) in A2780-CisR, TOV21G and SKOV3 cells respectively (Fig. 1d, Additional file 2: Figure S1B&C). However, such increase in *PIK3CA* expression was not observed in their respective NSP fractions.

### DNA-protein pull down identified critical *PIK3CA* promoter binders in platinum resistant cells

To identify the potential regulators, we adopted a magnetic bead-separation methodology where DNA-binder proteins were pulled down using biotinylated *PIK3CA* promoter as a bait. Since pulldown with the full-length *PIK3CA* promoter (1 kb) was not feasible due to obvious multi-target complexity, a smaller promoter fragment was used. To select the appropriate fragment of *PIK3CA* promoter, promoter activity of all four deletion constructs each with p53 binding site were assessed (Fig. 1f). The 5′-*PIK3CA* deletion construct (OS4-*PIK3CA*) exhibited maximal promoter activity (1.5–2.4 fold) in resistant (A2780-CisR, TOV21G, and SKOV3) cells compared to the full-length promoter, which was further increased (~1.3 fold) upon cisplatin treatment (Fig. 1g, Additional file 2: Figure S1D&E). Bioinformatic analysis with JASPER revealed presence of 116 putative transcription factor-binding sites in OS4, majority of which are also present on the full-length promoter sequence.

**Fig. 2:**
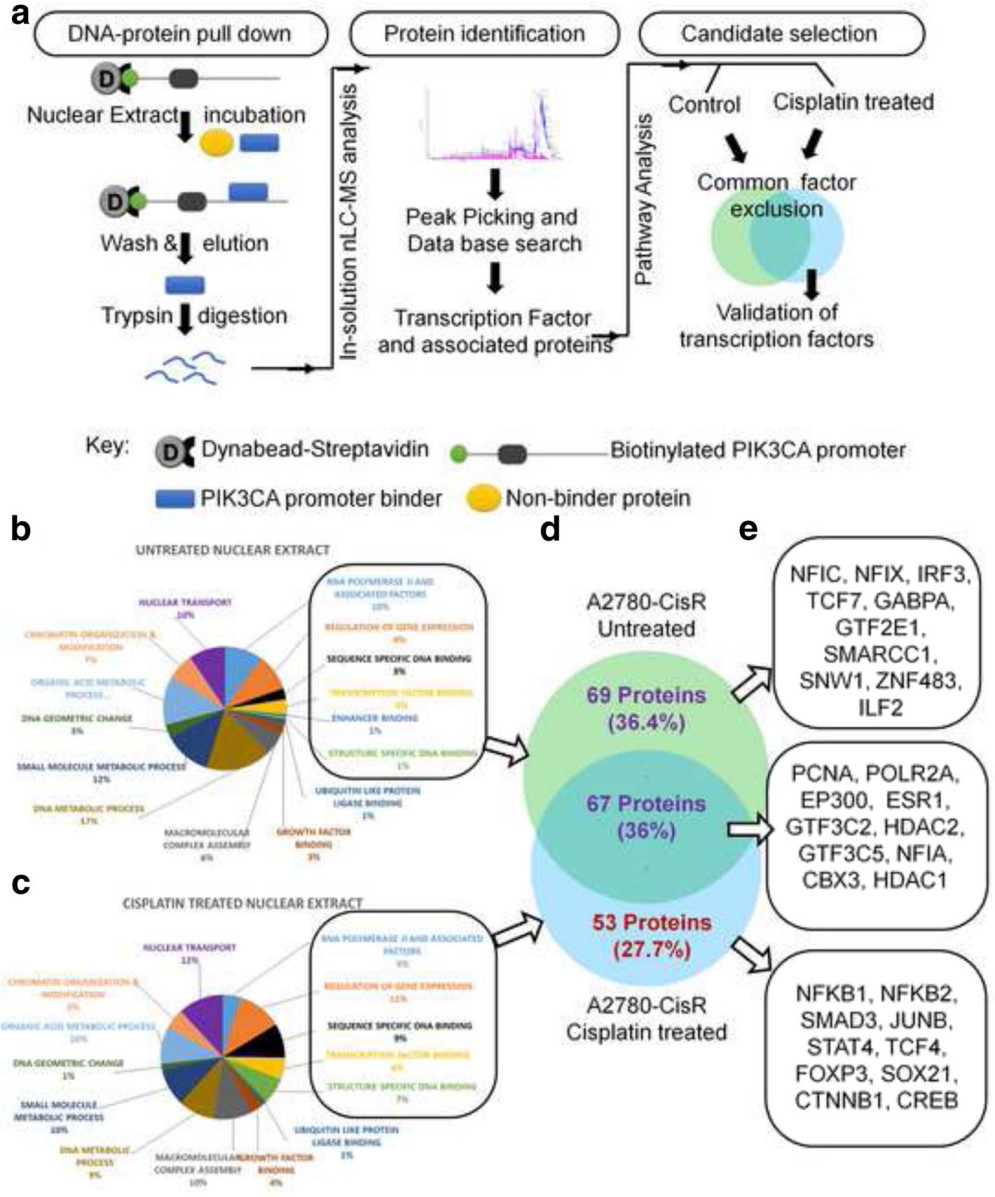
Identification of cisplatin responsive *PIK3CA* regulator in resistant cells: **a** Flow-chart of DNA-protein pulldown assay performed with nuclear extracts of pre and post cisplatin treated A2780-CisR cells. **b**&**c.** Identified protein signatures were functionaly categorised using GSEA server. Only transcription factors and their associated partner proteins were used for further analysis. **d**&**e.** potential binders were grouped from mutually inclusive or exclusive hits from untreated and treated A2780-CisR cells using Venn 2.1.0 server. Ten top scoring factors from each category were listed in the representative boxes. The complete list is given in the Additional file 1: Table S1-S3

**Fig. 3:**
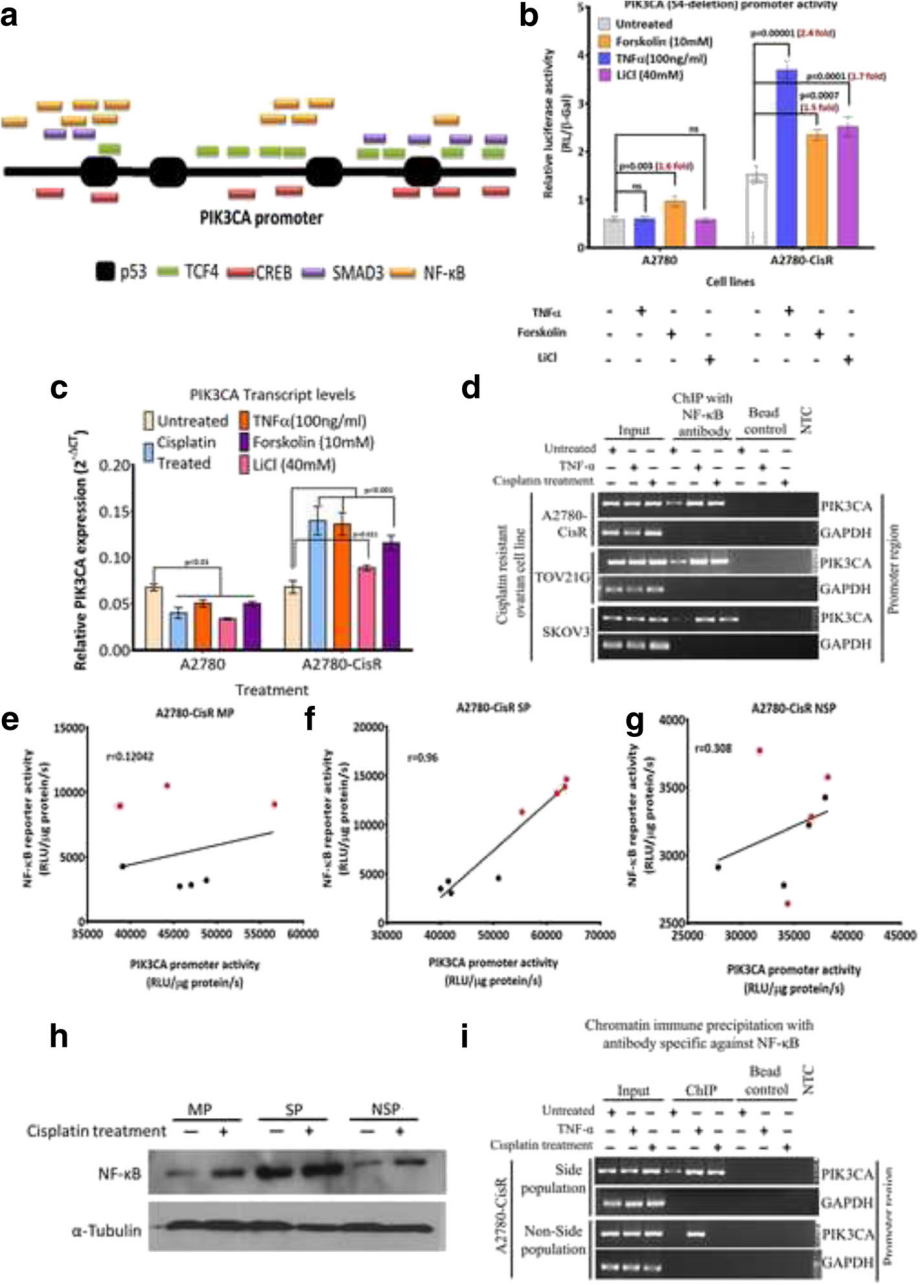
Cisplatin responsive activation of NF-κB induced *PIK3CA* in SP fraction of resistant cells. **a** Schematic of predicted response elements of five transcription factors on full-length *PIK3CA* promoter identified using JASPER server. **b** TNFα and LiCl treatment increased OS4-*PIK3CA* promoter activity only in A2780-CisR but not in A2780. Forskolin induced marginal change in both cells. Fold change (treated/control) were represented in red font (*n* = 4). **c** qPCR showed induction in *PIK3CA* expression only in A2780-CisR but not in A2780 cells after TNFα, LiCl and forskolin treatment. **d** Both cisplatin and TNFα augmented interaction of NF-κB on *PIK3CA* promoter as assessed by ChIP assay in A2780-CisR, TOV21G, and SKOV3 cells. **e**-**g** Perason correlation analysis showed a strong positive correlation between NF-κB reporter and *PIK3CA* promoter activity only in SP but not in MP or NSP cells in response to cisplatin. Black -untreated and red -treated. (Note- overlapping values in cisplatin treated MP are appeared as a single dot) (*n* = 4). **h** Protein levels of NF-κB pre and post cisplatin treatment. **i** ChIP assay for NF-κB occupancy on *PIK3CA* promoter in SP and NSP after TNFα and cisplatin treatment. GAPDH was used as control for ChIP experiments. Data represented as average ± SEM of four independent replicates with their actual p-value for statistical significance (ns-non significant). Also see Additional file 3: Figure S2, Additional file 4: Figure S3 and Additional file 5: Figure S4

Using DNA-protein pull down assay, putative binders were isolated from nuclear extracts of A2780-CisR cells (control or cisplatin treated) and processed for nLC-MS analysis. These MS/MS spectra were analysed by protein pilot/Mascot server and identified 312 and 246 (cisplatin treated) and 863 and 339 (untreated) proteins in two biological replicates of A2780-CisR cells. We clustered protein signatures from each independent replicates of untreated and cisplatin treated A2780-CisR cells based on their ‘GO- biological and GO- molecular’ functions using GSEA (Fig. 2a-c). Pathway analysis of the identified hits revealed 136 (untreated) and 120 (treated) protein signatures comprised of transcription factors and their associated factors. Protein overlap analysis using VENN 2.1.0 sever revealed 67 common binders (36% of total hits) in both untreated and cisplatin-treated A2780-CisR cells (Fig. 2d) which comprised of general transcriptional assembly including RNA pol-II, PCNA and EP300 (Fig. 2e). List of these identified proteins is given in Additional file 1: Table S1–3. Apart from these overlapping *PIK3CA* promoter binders, we found an exclusive set of 69 (36.4%) and 53 (27.7%) hits in untreated and cisplatin treated A2780-CisR cells respectively. These proteins were further clustered based on a) presence in both replicates; b) presence of their interacting partners in replicates and c) predicted binding sites on *PIK3CA* promoter (Fig. 3a). The top three promoter binders found to be NF-κB, β-catenin and CREB which are known to maintain self-renewal and differentiation of CSCs in ovarian (NF-κB) [4], nasopharyngeal (β-catenin) [18] and oral cancer (CREB) [19] cells.

### Cisplatin mediated induction of NF-κB transcriptionally activated *PIK3CA* expression in resistant cells

To compare the specificity of *PIK3CA* regulation by NF-κB, β-catenin and CREB, we assessed promoter activity of OS4-*PIK3CA* in A2780 and A2780-CisR cells after TNFα, Lithium chloride (LiCl) and forskolin treatment which are known inducers of these factors. Treatment of TNFα and LiCl increased OS4-*PIK3CA* promoter activity by 2.4 and 1.7 fold in A2780-CisR cells but not in sensitive A2780 cells (Fig. 3b). However, forskolin induced OS4-*PIK3CA* promoter activity by 1.5 fold in both A2780 and A2780-CisR cells (Fig. 3b) indicating a more general transcriptional regulation. Similar trend was also observed for full-length *PIK3CA* promoter activity (data not shown). In accordance with promoter activity, TNFα and LiCl escalated endogenous *PIK3CA* expression by 2.2 and 1.7 fold in A2780-CisR but not in A2780 cells (Fig. 3c). However, forskolin enhanced *PIK3CA* expression only in A2780-CisR (1.5 fold) but not in A2780 cells (0.7 fold) (Fig. 3c).

Collectively our data point towards a critical role of NF-κB in PI3K/AKT signalling in platinum-resistant cells. Next, to study the SP-specific *PIK3CA* regulators, we assessed *PIK3CA* promoter activity in SP fraction after treatment of cisplatin, TNFα, LiCl or forskolin. Promoter activity was induced by cisplatin (1.5 fold) and TNFα (1.9 fold) but not by LiCl (1.1 fold) and forskolin (0.9 fold) (Additional file 3: Figure S2). This was also corroborated with NF-κB binding on *PIK3CA* promoter, where cisplatin and TNFα enhanced NF-κB occupancy from 1% to 5% and 3.8% of input in A2780-CisR SP fraction. Similarly, TOV21G and SKOV3 cells showed increased NF-κB binding from 0.3% to 6% and 0.1% to 3% post TNFα and from 0.3% to 3.4% and 0.1% to 2.7% post cisplatin respectively (Fig. 3d).

### Differential activity of NF-κB was responsible for discrepant *PIK3CA* expression in SP and NSP fraction

To understand NF-κB driven *PIK3CA* promoter regulation, we measured NF-κB transcriptional activity and *PIK3CA* promoter activity in MP, SP and NSP fraction of A2780-CisR dual reporter cells [20]. This dual reporter cells expresses a NF-κB transcriptional reporter (4xNF-κB binding sites driving a hRL-eGFP) along with the *PIK3CA* promoter sensor (*PIK3CA* promoter driving fl2-tdt). SP showed increase in NF-κB reporter activity after cisplatin (3.5 fold) or TNFα (4.8 fold) treatment (Additional file 4: Figure S3A). Correspondingly, cisplatin or TNFα escalated *PIK3CA* promoter activity by 1.5 and 1.8 fold in SP cells, respectively (Additional file 4: Figure S3B). Only MP but not NSP showed augmented NF-κB reporter and *PIK3CA* promoter activity after TNFα treatment. Though cisplatin induced NF-κB transcriptional activity (2.8 fold), no significant difference was observed in *PIK3CA* promoter activity in MP cells (Additional file 4: Figure S3). Further, statistical analysis showed a strong positive correlation between NF-κB reporter activity and *PIK3CA* promoter activity in SP cells (Pearson correlation, *r* = 0.96). Such correlation was not observed in MP and NSP cells (*r* = 0.12 and *r* = 0.3 respectively) (Fig. 3e-g).

**Fig. 4:**
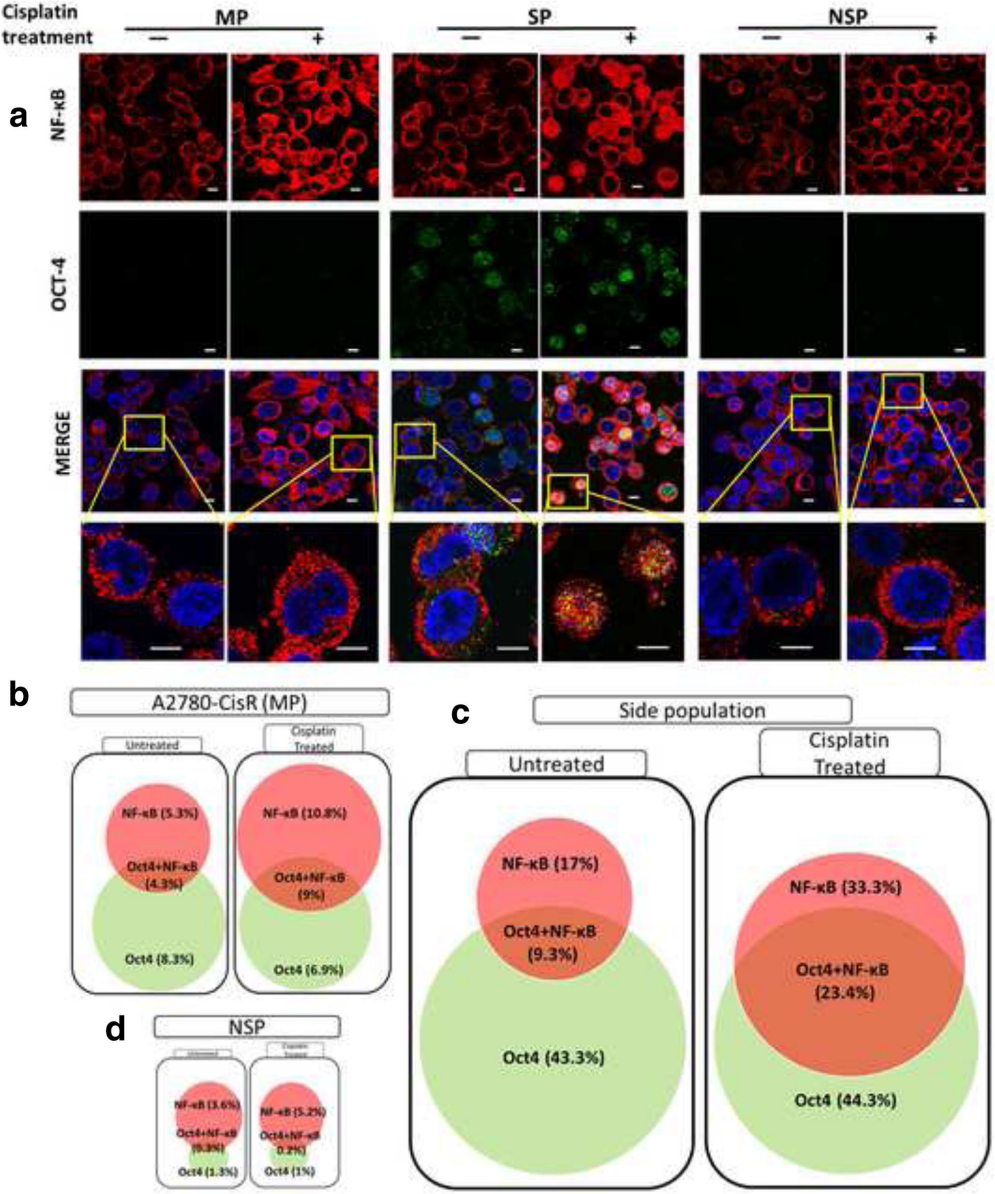
Cisplatin induced NF-κB translocation to nucleus especially in OCT4 positive cells: a Immunofluorescence for NF-κB (Red, first panel) and OCT4 (green, second panel) pre and post cisplatin treatment in MP, SP and NSP of A2780-CisR. Third (merged) and fourth (zoomed image) panel represents overlap of all three channels (blue-DAPI). (Scale-10 μm) **b**-**d** Quantification of NF-κB revealed significant induction in nuclear NF-κB only in MP (**b**), and SP (**c**) but not in NSP (**d**) upon cisplatin treatment. Cisplatin did not altered percentage of OCT4+ cells in all three fractions, however, it dramatically increased dual (NF-κB+ and OCT4+) cells in SP (**c**). Nuclear fluorescence for NF-κB (Red) and OCT4 (Green) was quantified from minimum of ~100 cells and represented as percentage ± SEM of single or dual staining. Also see Additional file 6: Figure S5

Cisplatin treatment induced total NF-κB level in MP, SP and NSP fraction compared to their untreated counterparts. Notably, control SP showed highest NF-κB level compared to MP and NSP cells (Fig. 3h). Further, NF-κB binding to *PIK3CA* promoter in untreated SP cells (0.9%), was increased up to 5.7 and 4.3% post TNFα or cisplatin treatment (Fig. 3i). In accordance with this and to our surprise, enhanced binding of NF-κB upon *PIK3CA* promoter by TNFα (0% to 2.2%) but not by cisplatin (0% to 0%) was observed in NSP fraction of A2780-CisR cells (Fig. 4g). TOV21G and SKOV3 also showed similar results with physical-interaction of NF-κB with *PIK3CA* promoter (Additional file 5: Figure S4A-C).

To understand CSC specific NF-κB activation, we performed co-localization studies for NF-κB and OCT4 in MP, SP and NSP fraction. Cisplatin treatment to A2780-CisR increased nuclear NF-κB+ cells from 5.2% to 10.8% (MP) and 17% to 33.4% (SP) (Fig. 4a-d). Similarly, TOV21G and SKOV3 cells showed ~2 fold increase (from 7.8% to 14.5% (MP) and 18.3% to 27.3% (SP) in TOV21G, 4.5% to 8.4% (MP) and 13.2% to 21.1% (SP) in SKOV3) in nuclear NF-κB+ cells (Additional file 6: Figure S5A-D). Nuclear NF-κB+ cells in NSP fraction remained unchanged after cisplatin treatment in A2780-CisR (3.8%), TOV21G (5.8%) and SKOV3 (3.4%) cells (Fig. 4d, Additional file 6: Figure S5A-D). Further, we quantified nuclear OCT4+ cells to detect CSC fraction from the MP, SP and NSP fraction. As expected, only SP obtained from A2780-CisR (44.3%), TOV21G (36.7%) and SKOV3 (32.4%) cells showed OCT4+ nuclei and cisplatin treatment did not alter their percent OCT4 positive cells (Fig. 4b-d, Additional file 6: Figure S5E&F). Notably, SP fraction of A2780-CisR, TOV21G and SKOV3 cells possessed 9.3%, 10% and 9% cells with NF-κB+ and OCT4+ nuclei respectively (Fig. 4, Additional file 6: Figure S5G&H). Contrarily, MP showed only 4.3%, 2.3%, and 1.3% cells with OCT4+ and NF-κB+ nuclei, while NSP fractions did not show any OCT4+ and NF-κB+ nuclei in A2780-CisR, TOV21G and SKOV3 respectively (Fig. 4b, d, Additional file 6: Figure S5G&H). These dual positive (OCT4+ and NF-κB+) nuclei were further increased up to 23.4%, 14% and 15% post cisplatin treatment in SP obtained from A2780-CisR, TOV21G and SKOV3 cells (Fig. 4c, Additional file 6: Figure S5G&H). Only MP fraction from A2780-CisR cells demonstrated increase in OCT4+ and NF-κB+ cells up to 9% (Fig. 4b), however, such increase was not observed in TOV21G (2.6%) and SKOV3 (2%) cells after cisplatin treatment (Additional file 6: Figure S5G&H). OCT4+ and NF-κB+ cells remained unchanged in NSP of in all three cells (Fig. 4d, Additional file 6: Figure S5G&H).

### Cisplatin triggered enrichment of cancer stem cells in *PIK3CA* dependent manner

**Fig. 5:**
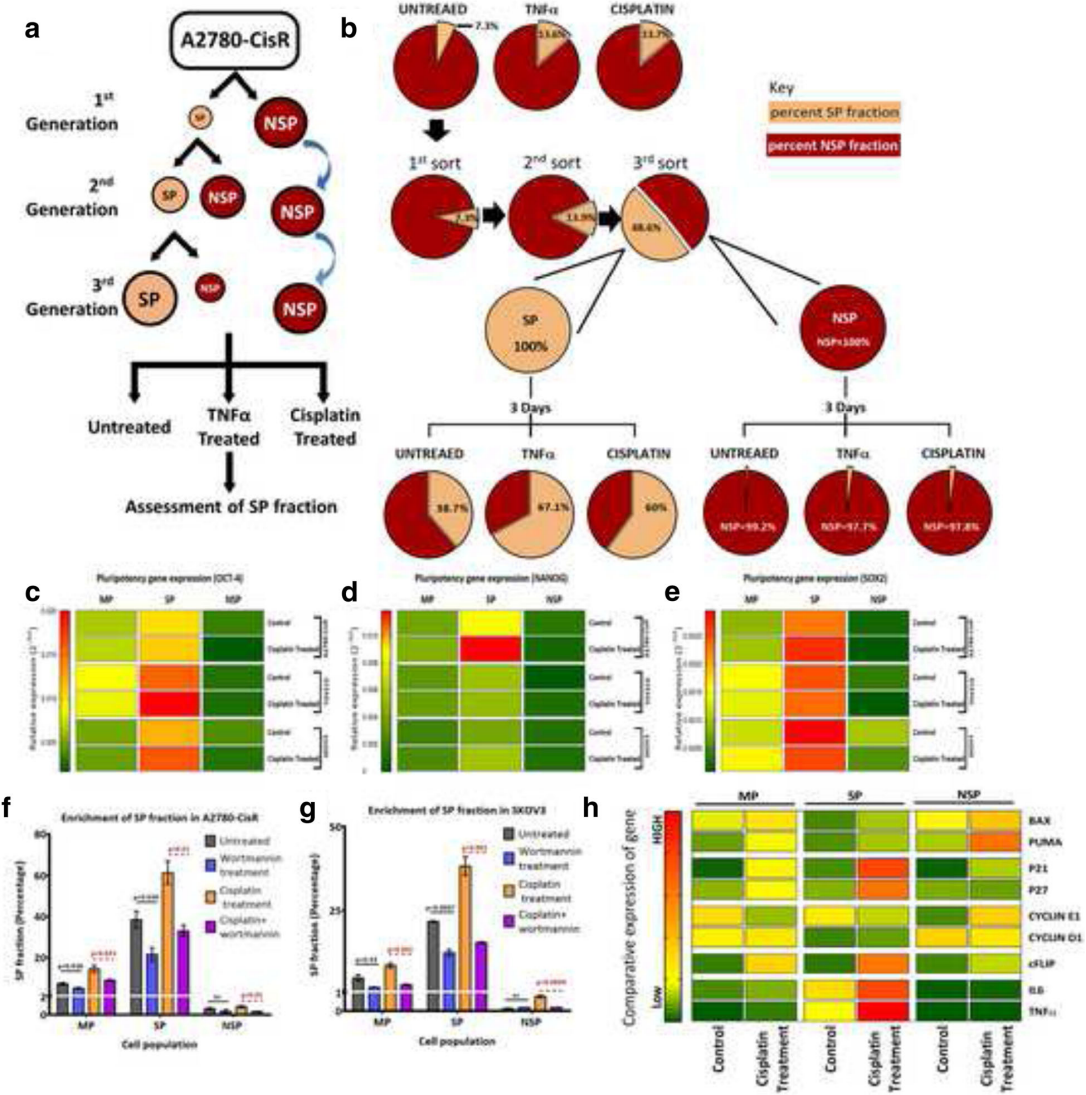
Cisplatin mediated activation of PI3K/AKT endorsed an anti-apoptotic, dormant state, augmenting CSC in resistant cells. **a** Flow-chart of SP-differentiation assay, where SP was serially sorted for three successive generation and %SP was determined pre and post TNFα or cisplatin treatment (24 h). **b** Pie chart showing SP enrichment after TNFα or cisplatin in A2780-CisR cells (MP, top panel). The middle panel depicted the percent increase in SP over generations as described in A and the lower panel depicted %SP after respective condition. The NSP cells did not showed any SP fraction. Data represented as average %SP obtained from three independent biological replicates. **c**-**e.** Heat map of relative expression (2^-ΔCT^) of pluripotent genes *OCT4* (**c**), *NANOG* (**d**) and *SOX2* (**e**) in response to cisplatin in MP, SP and NSP of A2780-CisR, TOV21G and SKOV3 cells (*n* = 3). **f**&**g.** Inhibition of *PIK3CA* activity using wortmannin decreased %SP in MP and 3rd gen SP of control and cisplatin treated A2780-CisR and SKOV3 cells (*n* = 3). **h** Comparative expression of the indicated genes in MP, SP and NSP fraction of A2780-CisR cells with and without cisplatin treatment where the least relative expression (2^-ΔCT^) value was considered be the lowermost color (dark green) of the scale. Data represented as mean of at least three independent replicates, and heat maps were generated with GraphPad prism 7. Also see Additional file 7: Figure S6

**Fig. 6:**
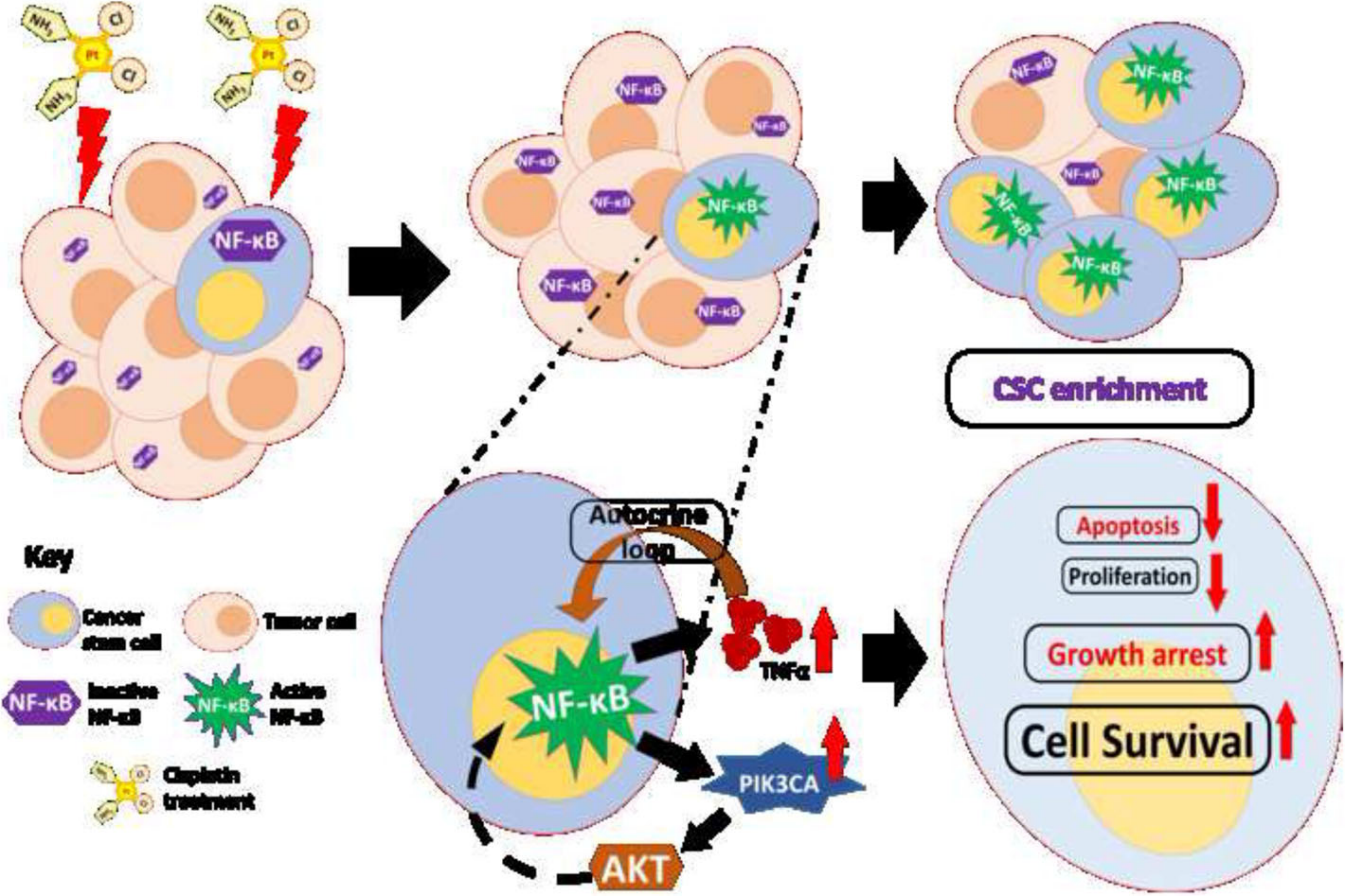
Proposed model of cisplatin induced CSC enrichment during cisplatin resistance. In platinum resistant cells, cisplatin mediated NF-κB activation only in cancer stem cells, which in turn activates bimodal feedback loop of NF-κB-TNFα and NF-κB-*PIK3CA*. In one hand, it promotes an autocrine loop by activating TNFα-NF-κB in CSC’c, and on another hand, it increases *PIK3CA* and PI3K/AKT signalling thus leading to NF-κB stabilisation. Activated PI3K/AKT confers resistance against cisplatin action through modulation of anti-apoptotic (increase in *cFLIP*) and pro-apoptotic (decrease in *BAX* and *PUMA*) genes. A constant supply of NF-κB through TNFα-NF-κB autocrine loop and enhanced stabilisation of NF-κB by activated AKT maintains an anti-apoptotic, quiescent CSC state confers their survival against chemotherapeutics in resistant cells

To understand the impact of *PIK3CA* expression modulation on the CSC-phenotype, we assessed enrichment of SP fraction in resistant cells post induction of *PIK3CA* using TNFα or cisplatin. Similar to cisplatin treatment (Fig. 1c), TNFα also augmented SP fraction from 7.3 ± 0.7% to 13.6 ± 1.9% in A2780-CisR cells (Fig. 6a, b). In other resistant cells, SP fraction increased from 5.9 ± 0.7% to 9.1 ± 1.6% (TOV21G) and 4.9 ± 0.7% to 7.7 ± 0.8% (SKOV3) post TNFα (Additional file 7: Figure S6A&B). To determine whether cisplatin plays a role in maintenance and/or differentiation of CSC fraction, we repeatedly sorted SP from resistant cells and cultured for three generation as reported earlier [17] (Fig. 5a). We observed that, from 100% of A2780-CisR sorted SP fraction, only 38.7 ± 3.5% of population persisted as SP fraction over 48 h while rest of the cells differentiated into NSP fraction (Fig. 5b). Interestingly, addition of TNFα or cisplatin in the media led to increase in SP fraction up to 67.1 ± 7.2% and 59.9 ± 12.2% respectively (Fig. 5b). Similar enrichment in SP fraction was also observed in TOV21G (31.5 ± 1.3% to 45.8 ± 7.9% by TNFα and 31.5 ± 1.3% to 46.6 ± 3.9% by cisplatin) and SKOV3 (25.1 ± 3% to 33.6 ± 3.1% by TNFα and 25.1 ± 3% to 33.6 ± 3.1% by cisplatin) cells (Additional file 7: Figure S6). NSP fraction from all three cells did not show any enrichment in SP fraction with either of the treatments (Fig. 5b, Additional file 7: Figure S6). To confirm whether this enrichment of SP fraction also enriches CSC-characteristics, we assessed expression of pluripotency factors (*OCT4*, *NANOG* and *SOX2*) pre and post cisplatin treatment. Cisplatin had also increased one or more pluripotency gene signatures in SP fraction of A2780-CisR (*NANOG*, *SOX2*), TOV21G (*OCT4*), and SKOV3 (*OCT4, NANOG*) (Fig. 5c-e). MP and NSP fraction did not show such alteration.

### Inhibition of *PIK3CA* activity suppresses cisplatin induced SP enrichment in platinum resistant cells

We observed relatively higher induction of *PIK3CA* expression in SP compared to MP and NSP upon cisplatin treatment, which also persuaded enrichment of SP fraction in A2780-CisR, TOV21G and SKOV3 cells. To ascertain that this enrichment depends on *PIK3CA* activity, we inhibited PI3K-activity using a reversible PI3K inhibitor, wortmannin. PI3K inhibition reduced SP fraction from 7.3 ± 1.1% (untreated) to 5.2 ± 0.6% (wortmanin) and 14.6 ± 2.9% (cisplatin) to 9.1 ± 0.8% (cisplatin and wortmannin) in A2780-CisR MP fraction (Fig. 5f). Similar effect was also observed in SKOV3 cells where SP fraction was reduced from 4.8 ± 1.7% (untreated) to 1.9 ± 0.2% (wortmannin) and 8.5 ± 1.2% (cisplatin) to 2.8 ± 0.4% (cisplatin and wortmannin) (Fig. 5g). Effect of PI3K inhibition was more robust in serially sorted 3rd generation SP where ~50% decline in SP-enrichment was evident post wortmannin treatment (24 h). In 3rd generation A2780-CisR cells, *PIK3CA* inhibition reduced SP from 38.5 ± 7.1% (untreated) to 21.6 ± 5.8% (wortmanin) and 61.3 ± 9.5% (cisplatin) to 33 ± 4.8% (cisplatin and wortmannin) (Fig. 5f). Similarly SP percentage of 3rd generation SP fraction obtained from SKOV3 cells was reduced from 21.7 ± 0.4% (untreated) to 12.3 ± 1.7% (wortmannin) and 38.3 ± 4.7% (cisplatin) to 15.4 ± 0.4% (cisplatin and wortmannin) (Fig. 5g). NSP cells did not show significant percentage of SP fraction after serial culturing with or without wortmannin treatment (Fig. 5f, g).

To assess the consequence of activated PI3K/AKT cascade on CSCs, we monitored expression of specific downstream targets such as *P21*, *P27*, *CYCLIN-D1* & *CYCLIN-E1* (cell cycle regulators), *BAX*, *PUMA* (pro-apoptotic) and *cFLIP* (anti-apoptotic) in MP, SP and NSP fractions pre and post cisplatin treatment. Increased *P21* and *P27* levels and decreased *CYCLIN-D1&*E1 levels in SP fractions compared to NSP cells marks growth-arrested, quiescent state of SP (Fig. 5h). Interestingly, we observed sharp increase in *CYCLIN-D1* level indicating active proliferation of NSP cells after cisplatin treatment. Though *cFLIP* levels were increased in all three fractions, transcriptional increment in *BAX* and *PUMA* in NSP fractions marked their pro-apoptotic fate (Fig. 5h). However, increased *cFLIP* levels along with quiescent-state of SP aid these cells to survive the action of chemotherapeutics. In accordance with NF-κB-transcriptional reporter activity, its downstream targets (*IL6* and *TNFα*) also showed higher expression in SP fractions compared to MP and NSP, which further increased significantly upon cisplatin treatment in MP and SP but not in NSP. However, *CYCLIN-D1*, another target of NF-κB did not show similar modulation in SP fractions. Overall, anti-apoptotic, dormant state provided survival advantage to CSC against cisplatin cytotoxicity and led to their enrichment upon cisplatin treatment (Fig. 6).

## Discussion

Chemoresistance, either intrinsic or acquired, substantially handicaps the efficacy of chemotherapy, escalating mortality rates in cancer patients. Acquirement of resistance towards chemotherapeutics is a dynamic and multifactorial process and influenced by cancer stem cell (CSC) enrichment [3, 21, 22]. Though platinum resistance is a common problem for cervical, head and neck, and non-small-cell lung cancer management, it is particularly devastating for epithelial ovarian cancer (EOC) patients as 50% of the therapy-responders ultimately show chemoresistant-relapse [7, 23]. Utilizing A2780-cisplatin resistant cellular model and two naturally occurring cisplatin resistant cells (TOV21G and SKOV3) [14], we attempted to address a unique question of how cisplatin impact existing CSCs in resistant cells. In all these cells, cisplatin enhanced the inherently drug resistant side-population with increased *PIK3CA* expression. Activation of PI3K/AKT pathway is known to favour cellular endurance against chemotherapeutics and maintenance of CSC-population [2, 18] but regulators of this pathway in drug-resistant cells are yet to be identified. Using transcription-factor pulldown assay, we identified NF-κB, β-catenin and CREB as cisplatin-responsive transcriptional activators of *PIK3CA* promoter. Amongst them, NF-κB differentially activated *PIK3CA* only in CSC-enriched side-population but not in non-SP. Both cisplatin and TNFα induced interaction between NF-κB and *PIK3CA* promoter. Further, inhibition of PI3K-activity dramatically reduced this CSC-enriched SP fraction. Enhanced nuclear co-localization of NF-κB with OCT4 and augmented expression of *OCT4*, *SOX2* and *NANOG* in SP signified enrichment of CSC properties by cisplatin. Finally, gene expression analysis revealed that cisplatin mediated activation of PI3K/AKT led to an anti-apoptotic, dormant stage, which aided CSC to evade therapeutic action while actively proliferating non-CSC cells succumbed to cisplatin’s action. Intriguingly, TNFα, a well-known activator of NF-κB and an NF-κB–regulated gene showed enhanced expression following cisplatin treatment only in SP cells. Collectively, our data emphasize that therapeutic intervention to platinum-resistant ovarian cancer cells favours a predominant quiescent state in SP via an interdependent positive feedback loop between TNFα-NF-κB and PI3K/AKT signalling.

Cisplatin forms the first line therapy against malignancies including ovarian, breast and colorectal cancer, however, acquirement of resistance impaired therapeutic efficacy [6, 7]. Amongst several molecular determinants of cisplatin resistance, activated PI3K/AKT pathway turns out to be a key signalling that also aids in survival of CSCs [1, 2]. Lee et al., (2005) showed that escalated *PIK3CA* expression in OVCAR-3/CDDP (resistant) cells leads to inhibition of *BAX* translocation via activated PI3K/AKT conforming platinum-resistance [24]. Amplification of *PIK3CA* was associated with ovarian and uterine cervical carcinoma, which led to active PI3K/AKT signaling and resistance acquirement [25, 26]. However, mechanism for augmented *PIK3CA* expression is less dissected at molecular level. Intriguingly, we observed cisplatin itself could upregulate *PIK3CA* expression in resistant but not in sensitive cells [14]. Several transcription-factors (TF) such as p53/p73, cMyc, YB-1, CTF-2, ATF-4, ZNF143, mTFA, AP1, NF-κB, OCT1, SP1, β-catenin and CREB are known to be associated with cisplatin resistance, regulating expression of resistance associated genes [27, 28]. Among all these TFs, cisplatin directly influences transcriptional activities of p53, YB-1, ZNF143, mTFA, NF-κB, ATF-2, 3 and 4 [27]. We earlier reported that cisplatin-mediated phosphorylation of p53 at Ser46 promotes its binding, and repress *PIK3CA* promoter in sensitive cells. Absence of such phosphorylation in cisplatin-treated resistant cells resulted in loss *PIK3CA* attenuation [14] which, however, does not explain the apparent increase in *PIK3CA* expression level. Till now, only YB-1, NF-κB and FOXO3a are known to induce *PIK3CA* expression in unstressed condition [13]. No information is available on regulators of *PIK3CA* modulation in platinum-resistant cells in response to cisplatin. Thus to identify cisplatin-responsive regulators of *PIK3CA* promoter, we subjected *PIK3CA* promoter bound nuclear fraction (potential transcription-factors) from untreated and cisplatin treated A2780-CisR cells to nLC-MS. We used a short OS4-*PIK3CA* promoter fragment due to: A) it shows augmented promoter activity upon cisplatin treatment, B) Contains binding sites for p53, and other transcription-factors & C) to avoid multi-target binding complexity possible for full-length (1 kb) promoter. Multiple putative candidate proteins bound to *PIK3CA* promoter in untreated (863 and 339) and cisplatin treated (312 and 246) A2780-CisR cells were identified which were further classified based upon their transcription associated functioning. Among the binders present exclusively in cisplatin treated samples (36%), we selected the top three *PIK3CA* binders (NF-κB, β-catenin and CREB) for further validation. All these three transcription-factors are known to influence cisplatin resistance in various cancer cells. Li et al., (2016) demonstrated that cisplatin-mediated increase in β-catenin expression aids in resistance-development in oral squamous cell carcinoma [25, 29]. While CREB is shown to be activated by cisplatin in ovarian cancer cells [28] and hyperactivation of NF-κB is associated with cisplatin resistant human epidermoid carcinoma, KCP-4 cells [30]. Our earlier report also demonstrates that in absence of TLR4/MyD88 signalling, ovarian cancer chemoresistance is maintained by NF-κB transcriptional activation under cisplatin treatment [20]. Augmented *PIK3CA* expression in platinum resistant H460 cells was also supported with increased NF-κB and β-catenin but not in CREB levels compared to their parental counterpart [31]. In accordance with this, we observed augmented *PIK3CA* promoter activity only in platinum resistant cells upon treatment with TNFα (NF-κB inducer) or LiCl (β-catenin inducer), while, forksolin (CREB inducer) increased *PIK3CA* promoter activity in both sensitive and resistant cells. Though forskolin increased *PIK3CA* expression in A2780-CisR cells, its treatment to A2780 sensitive cells led to reduction in *PIK3CA* transcript levels. Other than CREB, forskolin is known to induce binding of Ets-2, phospho-p53 and AP1 TFs to MMP-2 promoter [32]. Hence, observed decrease in *PIK3CA* expression in A2780 cells may be influenced by other forskolin-induced TFs such as p53. Till date NF-κB’s role as a positive regulator for *PIK3CA* promoter in ovarian cancer cells by TNFα is known [33]. Herein, we demonstrate a unique mechanism of *PIK3CA* transcriptional upregulation by NF-κB upon cisplatin, a cytotoxic drug treatment in platinum-resistant cells. ChIP assay showed that NF-κB-*PIK3CA* promoter interaction increased several fold following TNFα or cisplatin treatment in A2780-CisR, TOV21G, and SKOV3 cells. Our data also suggest β-catenin complex to be another regulator of *PIK3CA* promoter, however, presence of only two interacting (SMAD3 & TCF4) partners of this complex among large numbers of identified binders and minimal (~1.5) fold increase in *PIK3CA* expression by LiCl did not suggest a robust role of this complex. Further experimental studies are required to elucidate the exact contribution of β-catenin complex.

Two distinct observations that cisplatin mediates SP-enrichment and increased NF-κB-*PIK3CA* promoter interaction, prompted us to assess the expression level of *PIK3CA* in MP, SP, and NSP fractions. Surprisingly, augmented *PIK3CA* expression in response to cisplatin found only in SP but not in NSP cells. Next, for comprehensive understanding of what regulates *PIK3CA* in SP, we treated SP cells with inducers of NF-κB, β-catenin or CREB. Among them, only TNFα augmented *PIK3CA* promoter activity in SP but not in NSP cells, indicating NF-κB as distinct activator of *PIK3CA* in SP cells. Further, to verify whether SP does contain transcriptionally active NF-κB, we used a dual-stable cellular system of A2780-CisR expressing NF-κB-transcriptional reporter and *PIK3CA* sensor [20]. Cisplatin treatment resulted in strong positive correlation between NF-κB activity and *PIK3CA* promoter activity only in SP but not in MP or NSP.

Nuclear factor-κB, (NF-κB) belongs to a pivotal transcription-factor family, which controls expression of diverse genes related to immune-response, survival, proliferation, angiogenesis, and metastasis. In most cells, inhibitor of κB, (IκB) regulates NF-κB transcriptional activity [34] and dissociation of NF-κB/IκB heterodimer leads to nuclear-translocation of NF-κB where it functions as transcription-factor. The NF-κB signalling cascade converges with several cellular pathways including PI3K/AKT, where activated AKT promotes IκB degradation via phosphorylating IKKα kinase. In most tumors, NF-κB signaling is constitutively active, regulating gene induction associated with proliferation (*CYCLIN-D1*&*D2*, *CDK2* and c-*MYC*), growth signals (*GM-CSF* and *IL6*), anti-apoptosis (c*FLIP, BCL2, Bcl-xL and IAPs*) and angiogenesis (*VEGF, TNFα,* and *IL1*) [35]. Further, ovarian CD44 + CSCs, survive the treatment of paclitaxel and carboplatin through concomitant activation of NF-κB signalling and conferring resistance against these drugs [4]. Hence, to understand the CSC-specific activation of NF-κB in cisplatin resistant cells, we investigated co-localisation of OCT4 with NF-κB in MP, SP and NSP. ~2–3 fold increase in nuclear NF-κB+ and OCT4+ cells in only SP but not in MP or NSP upon cisplatin treatment, clearly indicating contribution of NF-κB in both CSC-homeostasis and *PIK3CA*-regulation. Enhanced expression in pluripotency gene (*OCT4*, *SOX2* and *NANOG*) by cisplatin exclusively in SP but not in MP or NSP warranted escalation of CSC-characteristics in SP fraction. Interestingly, same drug treatment induced distinct pluripotent gene expression in A2780-CisR, TOV21G, and SKOV3 cells probably due to intercellular differences in genetic constituents. Further to inspect NF-κB mediated *PIK3CA* regulation, we assessed NF-κB-*PIK3CA* promoter interaction in SP and NSP cells pre and post TNFα or cisplatin treatment. Similar to MP, SP but not NSP showed enhanced occupancy of NF-κB on *PIK3CA* promoter following cisplatin treatment. TNFα, however, induced NF-κB binding on *PIK3CA* promoter in both SP and NSP. This seemingly contradictory NF-κB-*PIK3CA* promoter interaction by two different stimuli is not surprising as NF-κB activation by UV-C or doxorubicin is known to induce a complete different set of target genes than that by TNFα and produce entirely different functional consequences [9].

Self-renewal and differentiation are the two major characteristics of stem cells that influence and regulate organogenesis and normal development. Likewise, cancer stem cells control tumor development through self-renewal and differentiation into proliferating tumor cells. If a cytotoxic drug can enrich CSC-population in resistant cells, it would certainly affect one of these two crucial properties. Both cisplatin and TNFα were found to favour a self-renewing undifferentiated state of CSCs as evident from 1.7–2 fold enrichment of SP. In addition, our study revealed that cisplatin mediates CSC-specific activation of NF-κB, which in turn induces expression of *TNFα* and *PIK3CA*. This incremented TNFα is known to act as an autocrine cue in concomitant NF-κB activation [35, 36], while activation of NF-κB escalates *PIK3CA* in CSC’s. All these data led us to hypothesize that NF-κB controlled *PIK3CA* expression coordinates the CSC-survival and CSC-plasticity under the influence of a chemotherapeutics. Indeed, treating SP with wortamannin, an irreversible PI3K*-*inhibitor, with and without cisplatin diminished the SP fraction. Gene expression analysis showed increased *P21*, *P27* and *cFLIP* and decrease in *CYCLIN-D1* and *CYCLIN-E1* in cisplatin treated SP cells pointing towards an anti-apoptotic, quiescent phase. In contrast, augmented *CYCLIN-D1&E1* expression in cisplatin treated NSP cells marking their proliferative state make them vulnerable towards drug. Activation of PI3K/AKT confers resistance against cisplatin action through up regulation of anti-apoptotic genes such as *cFLIP*. Overall, CSC’s with active PI3K/AKT and NF-κB signaling acquire anti-apoptotic, quiescent state conferring survival advantage against action of chemotherapeutic drugs (Fig. 6).

## Conclusion

Platinum-salts are in mainstay of cancer clinic for last few decades and will be continued for decades due to their potent action against proliferating cells. Resistance acquirement against platinum-compounds is a major concern for several malignancies including epithelial ovarian cancer. The unanticipated finding of cisplatin inducing CSC-enriched side-population in platinum-resistant cells opened a new dimension in our understanding on how action of a cytotoxic drug be modulated by cellular ambience. Activated NF-κB leading to upregulated *PIK3CA* expression is the key molecular feature behind this action of cisplatin. Though involvement of NF-κB mediated other signalling cascades are possible, our present data signifies PI3K/AKT pathway as the major determinant of cisplatin action in resistant cells. Perturbation in CSC homeostasis through blocking PI3K/AKT signalling might become a rational approach to intervene Platinum-resistant relapse.

## Additional file

Additional file 1: Table S1. List of protein identified exclusively in A2780-CisR control (untreated) cells. **Table S2.** List of protein identified exclusively in cisplatin treated A2780-CisR cells. **Table S3.** List of protein identified mutually in untreated and cisplatin treated A2780-CisR cells (XLSX 26 kb)
Additional file 2: Figure S1. Cisplatin augmented PIK3CA expression in TOV21G and SKOV3 cells. A. Cell viability assay with sub lethal treatment of cisplatin (10 μg/ml) for 24 h revealed 44% decrease in cell viability in A2780, while resistant cells (A2780-CisR, TOV21G and SKOV3) cells showed ~16–24% reduction in cell viability (*n* = 4). B & C: Real time quantification of PIK3CA expression revealed treatment of cisplatin augmented level of PIK3CA expression in SP fraction but not in NSP fraction of TOV21G (B) and SKOV3 (C) cells (*n* = 3). D & E. Only site 4 but not 1, 2 and 3 containing deletion construct showed augmented PIK3CA promoter activity upon cisplatin treatment in TOV21G (D) and SKOV3 (E) cells after transient transfection. All data were represented as average + SEM of at least three independent biological replicates with their actual *p*-value for statistical significance (ns- non significant). (TIFF 360 kb)
Additional file 3: Figure S2. NF-κB escalated PIK3CA promoter activity in SP cells. Cisplatin and TNFα but not lithium chloride or forskolin increased PIK3CA promoter activity in SP collected from A2780-CisR cells (*n* = 4, p-value denoted significant statistical different, ns- non significant). (TIFF 400 kb)
Additional file 4: Figure S3. NF-κB drove PIK3CA expression in SP cells upon cisplatin treatment. A. Treatment of TNFα or cisplatin dramatically increased renila activity driven by NF-κB response elements only in MP and SP fractions of A2780-CisR dual reporter cell line but not in NSP fraction. TNFα treatment to NSP fraction showed minimal increase in NF-κB transcriptional activity (*n* = 4). B. Similar to NF-κB-reporter activity, TNFα treatment also induced PIK3CA promoter activity in MP, SP and NSP fractions of A2780-CisR dual reporter cell line. However, cisplatin mediated increase in PIK3CA promoter activity was observed only in SP fraction. SP fraction showed much higher induction of PIK3CA promoter activity compared to MP cells after TNFα, or cisplatin (*n* = 4). (TIFF 403 kb)
Additional file 5: Figure S4. Cisplatin induced NF-κB and its physical interaction with PIK3CA promoter in TOV21G and SKOV3 cells. A & B. Similar to A2780-CisR cells, both TOV21G (A) and SKOV3 (B) cells showed higher NF-κB protein levels in SP fraction compared to MP and NSP cells as assessed through immunoblotting. All three fraction from both the cell lines demonstrated cisplatin mediated increase in NF-κB protein levels. C. Similar to A2780-CisR cells, TNFα or cisplatin treatment induced NF-κB occupancy on PIK3CA promoter only in SP fraction of TOV21G and SKOV3 cells. In NSP fraction, only TNFα treatment showed NF-κB binding to PIK3CA promoter in both the cell lines. PCR using primers for GAPDH was used to show purity of chromatin immune-precipitation. (TIFF 357 kb)
Additional file 6: Figure S5. Cisplatin triggers nuclear localization of NF-κB in OCT4 positive SP cells. A-H. Confocal microscopy for NF-κB and OCT4 staining was performed pre and post cisplatin treatment in TOV21G (upper panel) and SKOV3 (lower panel) cells and quantification were graphically represented. A & B. Mean nuclear fluorescence intensity measurement demonstrated higher nuclear localization NF-κB in SP fractions of TOV21G (A) and SKOV3 (B) compared to their MP and NSP fractions. Intriguingly, nuclear localization of NF-κB was further increased upon cisplatin treatment in MP and SP fractions of both, TOV21G and SKOV3 cells. C-F. Cisplatin treatment increases only NF-κB nuclear positive cells but not OCT4 positive cells in MP, SP and NSP fractions obtained from TOV21G (C & E) and SKOV3 (D & F) cells. G & H. Similar to A2780-CisR, cisplatin increased NF-κB nuclear translocation in SP fractions, especially in OCT4 positive cells of TOV21G and SKOV3 cells. Only MP but not NSP fractions showed nominal increase in co-nuclear localised NF-κB and OCT4 positive cells upon cisplatin treatment. All the quantifications were performed from minimal of ~100 cells and data was represented as either mean + SEM or percent + SEM. *p*-value represents statistical significance (student t-test) and ns- no significant difference. (TIFF 513 kb)
Additional file 7: Figure S6. SP differentiation assay for evaluation of CSC enrichment in TOV21G and SKOV3. A & B. Quantification of percent SP fraction in demonstrated enrichment of SP fraction in MP (top panel) and SP (middle panel) fractions but not in NSP (lower panel) fractions after TNFα or cisplatin treatment compared their untreated counterpart. Represented percentage SP fraction was obtained from three independent biological replicates. (TIFF 240 kb)
Additional file 8: Supplementary Methods. (DOCX 23 kb)

## Abbreviations

CSC: Cancer stem cells
MP: Main population (whole population)
NF-κB: Nuclear factor kappa-light-chain-enhancer of activated B cells
NSP: Non side population (Non-CSC population)
PIK3CA: Phosphoinositide-3-kinase catalytic unit alpha
SP: Side population (CSC-enriched population)
TNFα: Tumor necrosis factor alpha
