# Supplementary material for: Cisplatin triggers cancer stem cell enrichment in platinum-resistant cells through NF-κB-TNFα-PIK3CA loop
Source: J Exp Clin Cancer Res. 2017 Nov 23;36:164. doi: 10.1186/s13046-017-0636-8 (PMC5701448; doi:10.1186/s13046-017-0636-8)
Supplement: Additional file 8: — Supplementary Methods. (DOCX 23 kb) [file 13046_2017_636_MOESM8_ESM.docx]

**Cisplatin triggers cancer stem cell enrichment in platinum-resistant cells through NF-κB-TNFα-PIK3CA loop**

Bhushan Thakur^1,2^, and Pritha Ray^1,2,^*

**Methods:**

**Reagents and Plasmids:**

Chemicals used in this study are enlisted in supplementary table 4. The PIK3CA promoter sensors, PIK3CA*-*hrl-egfp, PIK3CA deletion constructs, and normalisation vectors (CMV-fl2-tdt and CMV-β-galactosidase) are previously reported. ([Thakur and Ray, 2016](#_ENREF_31))

**Western blot:**

Western blotting was performed as described earlier. ([Gaikwad et al., 2015](#_ENREF_12)) Primary antibodies (β-actin (cat#A5316), and anti-mouse (cat#A5316) and anti-rabbit (cat#A0545) secondary antibodies were purchased from Sigma-Aldrich. NF-κB (p65) (cat#AB7970) antibody were procured from ABCAM respectively.

**Quantitative RT-PCR**

cDNA was synthesised using 2µg of total RNA extracted from cells and qRT-PCR was performed using SYBR-Green method. The relative expression levels of mRNAs were calculated by the 2-∆Ct method with GAPDH as an internal control ([Gaikwad et al., 2013](#_ENREF_11)). Primer sequences used are listed in supplementary table S5.

**Immunofluorescence:**

Immunofluorescence studies were performed as described earlier. ([Gaikwad et al., 2015](#_ENREF_12)) Briefly, cells were fixed with 4% paraformaldehyde, permeabilized with 0.025% Triton-X and probed with NF-κB and OCT4 antibody for overnight at 4^0^C. Next day, after 2hr of incubation with secondary antibody, cells were counterstained with DAPI and images were captured with LSM 710 (Carl Zeiss) microscope. At least five representative fields are studied for NF-κB and DAPI staining.

**Promoter activity of PIK3CA promoter constructs:**

For PIK3CA promoter activity measurement, cells were transiently transfected with PIK3CA-hrl-egfp and normalization vector in 9:1 ratio and promoter activity was recorded as ratio of RL/β-gal or RL/FL. Activity of all the reporters were measured as described earlier.([Gaikwad et al., 2015](#_ENREF_12)) All transfection experiments were performed in quadruplets and repeated at least twice.

**MTT assays**

Cell viability was assessed using the standard thiazolyl blue tetrazolium bromide (MTT) method and percent viability was counted using the formula {[Absorbance ^(Test)^ ÷Absorbance ^(Control)^] ×100}. All experiments were performed at least three times.

**Table S4 Chemicals used for study.**

| Chemical | Catalogue No. | Company |
| --- | --- | --- |
| Cisplatin | P4394 | Sigma |
| G418 disulfate salt | G9516 | Sigma |
| SuperFect Transfection Reagent | 301307 | Qiagen |
| D-Luciferin | L-8240 | Biosynth |
| Coelenterazine, native | C-7001 | Biosynth |
| Wortmannin | W1628 | Sigma |
| Lithium chloride (LiCl) | 203637 | Sigma |
| Forskolin | F3917 | Sigma |
| TNFα | 570104 | BioLegends |
| RNease kit (RNA extraction kit) | 74104 | Qiagen |
| Superscript III (cDNA synthesis kit) | 11752050 | Thermo Fisher |
| SYBR® Green PCR Master Mix | 4309155 | Thermo Fisher |
| proteinase inhibitor cocktail | P8340 | Sigma |

**Table S5: Primer sequences for real time PCR**

| Gene Name | Forward primer | Reverse primer |
| --- | --- | --- |
| PIK3CA | TCAAAGGATTGGGCACTTTT | GCCTCGACTTGCCTATTCAG |
| BAX | TGGAGCTGCAGAGGATGATTG | GAAGTTGCCGTCAGAAAACATG |
| cFLIP | CCTAGGAATCTGCCTGATAATCGA | TGGGATATACCATGCATACTGAGATG |
| P27 | TGCAACCGACGATTCTTCTACTCAA | CAAGCAGTGATGTATCTGATAAACAAGGA |
| PUMA | ATGGCGGACGACCTCAAC | AGTCCCATGAAGAGATTGTACATGA |
| P21 | GGCAGACCAGCATGACAGATT | GCGGATTAGGGCTTCCTCT |
| GAPDH | TGCACCACCAACTGCTTAGC | GGCATGGACTGTGGTCATGAG |
| CYCLIN-D1 | TATTGCGCTGCTACCGTTGA | CCAATAGCAGCAAACAATGTGAAA |
| IL6 | GGTACATCCTCGACGGCATCT | GTGCCTCTTTGCTGCTTTCAC |
| TNFα | AGTCAGATCATCTTCTCGAACC | CACCAGCTGGTTATCTCTCAGC |
| OCT4 | GTGGAGAGCAACTCCGATG | TGCAGAGCTTTGATGTCCTG |
| NANOG | AAAGCTTGCCTTGCTTTGAA | AAGTGGGTTGTTTGCCTTTG |
| SOX2 | AACCCCAAGATGCACAACTC | GCTTAGCCTCGTCGATGAAC |
| cMYC | AATGAAAAGGCCCCCAAGGTAGTTATCC | GTCGTTTCCGCAACAAGTCCTCTTC |

**Supplementary figure legends:**

**Figure S1: Cisplatin augmented PIK3CA expression in TOV21G and SKOV3 cells. A.** Cell viability assay with sub lethal treatment of cisplatin (10μg/ml) for 24hrs revealed 44% decrease in cell viability in A2780, while resistant cells (A2780-CisR, TOV21G and SKOV3) cells showed ~16-24% reduction in cell viability. (n=4) **B & C:** Real time quantification of PIK3CA expression revealed treatment of cisplatin augmented level of PIK3CA expression in SP fraction but not in NSP fraction of TOV21G (B) and SKOV3 (C) cells. (n=3) **D & E.** Only site 4 but not 1, 2 and 3 containing deletion construct showed augmented PIK3CA promoter activity upon cisplatin treatment in TOV21G (D) and SKOV3 (E) cells after transient transfection. All data were represented as average + SEM of at least three independent biological replicates with their actual p-value for statistical significance (ns- non significant).

**Figure S2: NF-κB escalated PIK3CA promoter activity in SP cells.** Cisplatin and TNFα but not lithium chloride or forskolin increased PIK3CA promoter activity in SP collected from A2780-CisR cells. (n=4, p-value denoted significant statistical different, ns- non significant)

**Figure S3: NF-κB drove PIK3CA expression in SP cells upon cisplatin treatment. A.** Treatment of TNFα or cisplatin dramatically increased renila activity driven by NF-κB response elements only in MP and SP fractions of A2780-CisR dual reporter cell line but not in NSP fraction. TNFα treatment to NSP fraction showed minimal increase in NF-κB transcriptional activity. (n=4) **B.** Similar to NF-κB-reporter activity, TNFα treatment also induced PIK3CA promoter activity in MP, SP and NSP fractions of A2780-CisR dual reporter cell line. However, cisplatin mediated increase in PIK3CA promoter activity was observed only in SP fraction. SP fraction showed much higher induction of PIK3CA promoter activity compared to MP cells after TNFα, or cisplatin. (n=4)

**Figure S4: Cisplatin induced NF-κB and its physical interaction with PIK3CA promoter in TOV21G and SKOV3 cells. A & B.** Similar to A2780-CisR cells, both TOV21G (A) and SKOV3 (B) cells showed higher NF-κB protein levels in SP fraction compared to MP and NSP cells as assessed through immunoblotting. All three fraction from both the cell lines demonstrated cisplatin mediated increase in NF-κB protein levels. **C.** Similar to A2780-CisR cells, TNFα or cisplatin treatment induced NF-κB occupancy on PIK3CA promoter only in SP fraction of TOV21G and SKOV3 cells. In NSP fraction, only TNFα treatment showed NF-κB binding to PIK3CA promoter in both the cell lines. PCR using primers for GAPDH was used to show purity of chromatin immune-precipitation.

**Figure S5: Cisplatin triggers nuclear localization of NF-κB in OCT4 positive SP cells. A-H.** Confocal microscopy for NF-κB and OCT4 staining was performed pre and post cisplatin treatment in TOV21G (upper panel) and SKOV3 (lower panel) cells and quantification were graphically represented. **A & B.** Mean nuclear fluorescence intensity measurement demonstrated higher nuclear localization NF-κB in SP fractions of TOV21G (A) and SKOV3 (B) compared to their MP and NSP fractions. Intriguingly, nuclear localization of NF-κB was further increased upon cisplatin treatment in MP and SP fractions of both, TOV21G and SKOV3 cells. **C-F.** Cisplatin treatment increases only NF-κB nuclear positive cells but not OCT4 positive cells in MP, SP and NSP fractions obtained from TOV21G (C & E) and SKOV3 (D & F) cells. **G & H.** Similar to A2780-CisR, cisplatin increased NF-κB nuclear translocation in SP fractions, especially in OCT4 positive cells of TOV21G and SKOV3 cells. Only MP but not NSP fractions showed nominal increase in co-nuclear localised NF-κB and OCT4 positive cells upon cisplatin treatment. All the quantifications were performed from minimal of ~100 cells and data was represented as either mean + SEM or percent + SEM. p-value represents statistical significance (student t-test) and ns- no significant difference.

**Figure S6: SP differentiation assay for evaluation of CSC enrichment in TOV21G and SKOV3. A & B.** Quantification of percent SP fraction in demonstrated enrichment of SP fraction in MP (top panel) and SP (middle panel) fractions but not in NSP (lower panel) fractions after TNFα or cisplatin treatment compared their untreated counterpart. Represented percentage SP fraction was obtained from three independent biological replicates.
